# Flat Magnetic Stimulation for Urge Urinary Incontinence

**DOI:** 10.3390/medicina59111999

**Published:** 2023-11-14

**Authors:** Marta Barba, Alice Cola, Giorgia Rezzan, Clarissa Costa, Ilaria Re, Silvia Volontè, Stefano Terzoni, Matteo Frigerio, Serena Maruccia

**Affiliations:** 1 Department of Gynecology, IRCCS San Gerardo dei Tintori, University of Milano-Bicocca, 20900 Monza, Italy; m.barba8792@gmail.com (M.B.); alice.cola1@gmail.com (A.C.); g.rezzan@campus.unimib.it (G.R.); c.costa14@campus.unimib.it (C.C.); i.re2@campus.unimib.it (I.R.); s.volonte6@campus.unimib.it (S.V.); 2 Department of Urology, ASST Santi Paolo e Carlo, San Paolo Hospital, 20142 Milano, Italy; stefano.terzoni@asst-santipaolocarlo.it (S.T.); serena.maruccia@gmail.com (S.M.)

**Keywords:** overactive bladder, quality of life, urinary incontinence, pelvic floor disorders, magnetic stimulation

## Abstract

*Background and Objectives*: Strategies for overactive bladder syndrome (OAB) management involve, among others, strengthening the bladder outlet to suppress urgency and neuromodulating the sacral roots. Magnetic stimulation (MS) is a technology that involves an extracorporeal device that is able to provide an electromagnetic field specifically designed to interact with pelvic floor neuromuscular tissue. The resulting tissue electrical activity induces contraction of the pelvic muscle and neuromodulation of the S2–S4 sacral roots. Flat Magnetic Stimulation (FMS) is a relevant advancement involving homogeneous electromagnetic fields, which are able to optimize the effect on the entire pelvic area. However, the benefits of this new technology for OAB syndrome are poorly known. Consequently, the aim of our study is to analyze the outcomes and quality of life (QoL) impact of FMS with Dr. Arnold (DEKA, Calenzano, Italy) in women suffering from OAB syndrome associated with urinary incontinence. *Materials and Methods*: This prospective study included patients with OAB, urge urinary incontinence, and no ongoing OAB treatments. At baseline (T0), the Incontinence Impact Questionnaire (IIQ-7), the Female Sexual Function Index (FSFI-19), and the International Consultation on Incontinence Questionnaire–Urinary Incontinence Short Form (ICIQ-UI SF) were collected. Patients underwent 8 FMS sessions of 25 min each in one month. At the termination of the therapy (T1), women repeated the ICIQ-UI SF, FSFI-19, and IIQ-7 tools. Moreover, the Patient Global Impression of Improvement (PGI-I) questionnaire was collected to evaluate the cure rate. *Results*: Our study enrolled a total of 57 consecutive patients. Most women had at least one second- or third-line treatment before FMS, while the remaining naive patients had contraindications to pharmacological treatments. No women reported adverse effects during the treatment. After the treatment, we observed a decrease in the IIQ-7 (*p* < 0.001) and ICIQ-UI SF scores (*p* < 0.001) and an improvement in sexual function (*p* < 0.001) evaluated with FSFI-19. According to PGI-I scores, 42 (73.7%) women referred to some kind of improvement, scoring ≤ 3 points. Specifically, 8.7% of patients considered themselves very much improved, 29.8% much improved, 35.1% minimally improved, and 26.3% found no changes. FMS was effective in treating OAB symptoms without any adverse effects. The mechanism is supposed to be related to suppressing the initiation of micturition. This makes FMS a promising device for treating naive and refractory urge urinary incontinence. *Conclusion*s: The new FMS represents a promising non-pharmacological option for the treatment of naive and refractory OAB.

## 1. Introduction

Pelvic floor disorders are conditions affecting the proper function of a woman’s pelvic organs, thus encompassing bowel, urinary, support, and sexual dysfunctions [1,2]. Vaginal delivery and associated trauma are considered the primary etiopathogenetic mechanisms predisposing to pelvic floor damage [3,4,5]. Specific obstetric factors, such as fetal macrosomia and instrumental delivery, may be associated with greater pelvic floor injuries [6,7,8,9]. Moreover, menopause-related hormonal changes and connective tissue histological features have been related to the development of pelvic floor symptoms [10,11,12,13,14]. Due to shared risk factors, pelvic floor disorders may coexist, and worsening or de novo onset may occur after treatment such as surgery [15,16,17]. Overactive bladder syndrome, for instance, usually improves after prolapse repair but tends to worsen if a concomitant anti-incontinence procedure is performed at the time of surgery [18,19].

Overactive bladder syndrome (OAB) is defined by the International Continence Society (ICS) as “urgency, with or without urge incontinence, usually with frequency and nocturia” [20]. It is a common pelvic floor disorder, affecting a sixth of women in the United States [21]. Urinary incontinence has a negative impact on women’s quality of life (QoL) in terms of interpersonal, household, sexual, and mental-physical well-being [22,23]. OAB can be categorized, based on the presence or absence of neurologic conditions such as Parkinson’s disease, multiple sclerosis, and/or a spinal cord injury, into a neurogenic or idiopathic (nonneurogenic) form. However, the etiology of OAB is still unclear, but it has been related to bladder hypersensitivity, low bladder compliance, detrusor overactivity, or pelvic floor surgery [22,24]. Detrusor overactivity (DO) represents the most common finding in OAB patients and can be identified in 64% of patients during urodynamic evaluation [25,26]. However, urodynamics globally shows poor agreement between clinical symptoms and instrumental findings in the evaluation of bladder dysfunction [27,28,29]. Moreover, the importance of urodynamics for urinary incontinence diagnostic work-up is currently under debate due to inconsistent performance and a lack of consensus on definitions [30,31]. In addition, both OAB and DO have been found in concomitance with underactive bladder syndrome and detrusor underactivity, making proper patients’ condition classification and subsequently clinical management challenging [32,33]. ICS has recognized that symptoms of OAB may stem from diverse forms of dysfunction in the urethra and bladder [20]. Recent research has posited the existence of several subcategories within OAB [34,35], suggesting that various underlying mechanisms can elicit the sensation of urinary urgency. An increasing body of evidence in recent years has linked OAB with conditions such as subclinical dysfunction in the autonomic nervous system [36,37], metabolic syndrome [38,39,40], deficiencies in sex hormones [41], affective disorders [42], gastrointestinal functional issues [43,44], and alterations in urinary microbiota [45,46]. These factors could contribute to OAB and indicate that it might have a distinct pathophysiological basis within these diverse frameworks. Although the available data are limited, several emerging studies point to the potential benefits of categorizing OAB into these different phenotypes, which could lead to more informed treatment decisions and potentially better outcomes. These subtypes of OAB could not be necessarily exclusive as they might often overlap, and this fact could provide a strong rationale for considering combination therapy, which could target multiple underlying mechanisms and enhance the likelihood of achieving successful treatment [47].

Currently, the first-line treatment for idiopathic OAB is behavioral treatment, which involves three approaches: (1) lifestyle modification such as fluid management, a decrease in the intake of caffeine, alcohol, and acidic and spicy foods, and weight loss; (2) modification of bladder function induced by changing voiding habits, such as applying bladder training and delayed voiding; and (3) pelvic floor muscle training (PFMT) to strengthen the bladder outlet and suppress urgency, which may include active exercises for pelvic floor muscles, biofeedback, electrical stimulation, and magnetic stimulation (MS) [48,49,50]. Second-line treatment involves pharmacological therapy with antimuscarinics, beta-3 adrenergic receptor agonists, or the combination of both of these medications. Combination therapy with an anti-muscarinic and beta-3 adrenergic receptor agonist may be considered for patients refractory to monotherapy [50,51,52,53]. The third line is represented by onabotulinumtoxinA therapy, PTNS, or neuromodulation, and may be offered—after careful patient selection and appropriate patient counseling—in patients in whom previous line treatments have failed or are contraindicated [51,54,55]. However, since all second- and third-line treatments have side effects, drawbacks, and complications, behavioral therapies should be offered at first to all patients with OAB.

Despite most guidelines not recognizing the role of magnetic stimulation, its hypertrophic effect on the urethral rhabdosphincter may be useful in suppressing the initiation of micturition, similar to other forms of pelvic muscle strengthening treatments [56]. This technology involves an extracorporeal device that is able to produce a distinct electromagnetic field that engages with neuromuscular tissue located in the pelvic floor. The resulting electrical activity induces controlled depolarization of the nerves, resulting in pelvic muscle contraction and sacral S2–S4 root neuromodulation [57]. Although the Food and Drug Administration has approved magnetic stimulation for the treatment of urinary incontinence since 1998, limited research in the existing literature has evaluated the safety and effectiveness of this practice [58]. A recent systematic review of the effectiveness of magnetic stimulation for the treatment of urinary incontinence demonstrated a certain efficacy in terms of cure rate and improvement in QoL [59]. However, only 12 studies published between 2010 and 2020 were eligible, and most of them considered patients with stress urinary incontinence.

Recently, magnetic stimulator technology witnessed relevant advancements, which include Flat Magnetic Stimulation (FMS). This involves homogeneous rather than curved electromagnetic fields, which are able to optimize the effect on the entire pelvic area. Due to the equal distribution and intensity of stimulation, FMS allows large recruitment of muscle fibers without leaving areas of inconstant/suboptimal stimulation, leading to substantial advantages compared with standard magnetic stimulation treatment. The interaction between the magnetic field and the neuromuscular tissue induces electrical currents, which initiate neuronal cell depolarization, trigger muscle contractions, and improve blood circulation [60]. Depending on the frequencies of magnetic fields, either sacral S2–S4 root neuromodulation or urethral rhabdosphincter muscle fiber hypertrophy may be evoked, which represent two milestones in the treatment of overactive bladder syndrome. Specifically, a previous sonographic study has demonstrated significant urethral rhabdosphincter muscle hypertrophy consequent to FMS [56]. However, the benefits of this new technology for OAB syndrome are poorly known. 

Consequently, the aim of our study is to analyze the outcomes and QoL impact of FMS in women suffering from overactive bladder syndrome associated with urinary incontinence.

## 2. Materials and Methods

This prospective study was carried out at “Fondazione IRCCS San Gerardo dei Tintori” (Monza, Italy) from August 2022 to March 2023. In the gynecological outpatients, women underwent a clinical interview to evaluate the presence and severity of pelvic floor symptoms, such as bulging symptoms, lower urinary tract symptoms, pelvic pain, or fecal incontinence. All definitions conformed to IUGA/ICS terminology [20]. For each patient, a clinical urogenital examination was performed, and genital prolapse was staged in accordance with the Pelvic Organ Prolapse Quantification (POP-Q) system. Patients were included in the study if they had an overactive bladder with urge urinary incontinence and no active treatments at the moment of enrollment. Among the exclusion criteria we considered were pregnancy, age < 18 years old, weight > 160 kg, deficient Italian language knowledge, defibrillator or implanted pacemaker carriers, patients with ferromagnetic prostheses or neurostimulators, recent diagnosis of malignant tumors, deep venous thrombosis, acute inflammatory conditions, fever, recent fractures involving the body area to be treated, arrhythmia, or congestive cardiac failure, as previously stated [56]. At baseline (T0), all the patients received and completed the Incontinence Impact Questionnaire (IIQ-7), the Female Sexual Function Index (FSFI-19), and the International Consultation on Incontinence Questionnaire–Urinary Incontinence Short Form (ICIQ-UI SF) [61,62,63]. The IIQ-7 questionnaire is aimed at exploring the effect of urinary incontinence on patients’ everyday lives and relationships. It is made of seven items—with four options of answers each—to individually self-esteem the effect of urine leaking on quotidian activities in four areas: physical exercise (points #1 and #2), traveling (points #3 and #4), social interactions (points #5), and psychological well-being (points #6 and #7). This tool has been associated with an excellent level of acceptability, reliability, and validity based on standard psychometric tests and validated across different countries and cultures [63]. The ICIQ-UI SF questionnaire includes four questions dealing with the urine leaking frequency, the subjective amount of urine leaking, and its impact on everyday life. Only the first three items determine the total score, while the last one is designed to self-define the subcategory of urinary incontinence. The above-mentioned tool demonstrated significant rates of validity, accuracy, and sensitivity through the use of standard psychometric tests, and its validation has allowed the evaluation of the frequency, severity, and effect of urinary incontinence on QoL [61]. The FSFI-19 questionnaire is a self-reported tool aimed at investigating female sexual dysfunction. It consists of 19 questions, divided into six domains, exploring sexual function, which includes arousal, desire, lubrication, orgasm, satisfaction, and pain, with 5-point Likert answer scales. A cut-off of 26.5 points identifies patients with and without sexual disorders. This tool has systematically proven satisfying assessment characteristics in evaluating the impact of multiple conditions on sexual health and the efficiency of various therapies, representing one of the most appropriate, helpful, well-known, and powerful diagnostic tools in this field [62]. 

After obtaining written informed consent, patients underwent FMS according to the following schedule: eight sessions of 25 min each, two times a week for thirty days, of FMS treatment with Dr. Arnold (DEKA, Calenzano, Italy). The Dr. Arnold device is made of a comfortable and ergonomic chair with a built-in electromagnetic device controlled by an external main unit designed for deep pelvic floor area therapy and has been CE-marked since July 2020. The electromagnetic coil is located under the seat. The patient is seated in a manner that centers the perineum on the chair, ensuring that stimulation and muscle contraction are directed toward the pelvic floor. Before each session of FMS, an operator adjusted the patient’s position to both ensure appropriate electromagnetic stimulation and individual wellbeing throughout the entire treatment period. According to the standard position, the patients should have their legs bent at a 90-degree angle, with thighs parallel to the ground and feet resting flat on the floor; if necessary, the seat height could be adjusted. During sessions 1 to 4, the Hypotonia/Weakness 1 protocol was followed; this consisted of a primary phase of warm-up and gentle muscle activation, then a subsequent stage of muscular activity focused on restoring tropism and enhancing muscle tone within a trapezoidal pattern, involving frequencies in the range of 20–30 Hz, requiring a total time of 25 min.

During sessions 5 to 8, the Hypotonia/Weakness 2 protocol was followed; this included an initial phase for warming up and activating muscles, followed by a muscle-focused segment aimed at enhancing tropism (volume), and subsequently, a muscle strengthening phase (40–50 Hz) characterized by a trapezoidal pattern, requiring a total time of 25 min.

As treatment was fully completed (T1), women repeated all three questionnaires, the IIQ-7, ICIQ-UI SF, and FSFI-19 questionnaires, and the results were compared to the T0 to determine the impact on QoL. The Patient Global Impression of Improvement (PGI-I) questionnaire allowed researchers to investigate the subjective cure rate. This tool is a 7-point scale that provides a self-evaluation of how much the patient’s condition has improved or aggravated after the treatment, compared to the baseline status. This scale is rated as 1, very much improved; 2, much improved; 3, minimally improved; 4, no change; 5, minimally worse; 6, much worse; or 7, very much worse [64]. An improvement compared to the baseline (PGI-I score ≤ 3) was considered a success.

This study protocol (protocol code PF-MAGCHAIR) was approved by the local Ethics Committee. After the failure of the normality check, which was conducted with Shapiro–Wilk’s test, the questionnaire scores were presented as median values along with the interquartile range (IQR). Before comparing the results extracted throughout the analysis, we used the Mann–Whitney’s U test to verify if pertinent covariates such as patients’ age, number of deliveries, and body mass index could provide any statistically significant differences in the baseline scores. The scores were compared using Wilcoxon’s signed-rank test. We set the significance threshold at 0.05 for all calculations. The analysis was performed with R 4.1 (the R Core Team, Vienna, Austria, 2021) for MacOS^®^.

## 3. Results

Our study recruited 58 patients in total. One of them (1.7%) was lost at follow-up. The remaining 57 women were analyzed. Population characteristics are shown in Table 1. Most women had at least one second- or third-line treatment before FMS, while the remaining naive patients had contraindications to pharmacological treatments. The median age was 65 years, IQR [65;75], with 77.2% of the women having given birth to one (*n* = 15, 26.3%) or two (*n* = 29, 50.9%) children. A total of 14.0% were nulliparous (*n* = 8); the remaining five had three children (*n* = 3, 5.3%) or more children (*n* = 2, 3.6%).

No adverse effects have been documented by any of the patients during the whole procedure. A summary of outcome assessments from subjective and quality of life surveys is presented in Table 2, both at baseline (T0) and upon completion of the treatment (T1).

The reduction in the IIQ7 scores (leakage severity) at T1, compared to T0, was statistically significant (*p* < 0.001) with a mean difference of 13.2 points, 95%CI [8.7;17.3] and an effect size of 0.802, 95%CI [0.500–1.098], thus supporting the clinical usefulness of this treatment. Moreover, at T1, we observed a statistically significant reduction in the ICIQ-UI SF scores compared to T0 (*p* < 0.001) with a mean difference of 2.46 points, 95%CI [1.54; 3.37], and an effect size of 0.71, 95%CI [0.42–0.99]. Sexual function also improved significantly (*p* < 0.001) with a mean difference of −1.69, 95%CI [−2.59; −0.80], and an effect size of 0.504, 95%CI [0.23–0.78]. Considering sexually active women only, a significant improvement was observed in the IIQ scores (*p* < 0.001, mean difference 14.63 points, 95%CI [7.567–21.69], effect size 0.86, 95%CI [0.39–1.31]), ICIQ-UI SF (*p* = 0.003, mean difference 2.16 points, 95%CI [0.80–3.52], effect size 0.66, 95%CI [0.22–1.01]), and FSFI scores (*p* = 0.001, mean difference −3.30, 95%CI [−5.19; −1.42], effect size 0.72, 95%CI [0.28–1.16]).

**Figure 1:**
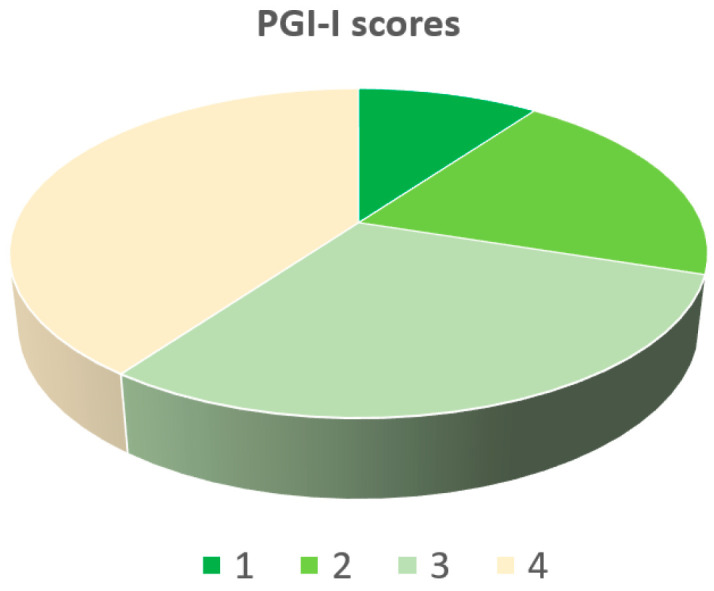
PGI-I scores.

According to PGI-I scores, 42 (73.7%) women referred to some kind of improvement, scoring ≤ 3 points. Specifically, 8.7% of patients considered themselves very much improved (1), 29.8% much improved (2), 35.1% minimally improved (3), and 26.3% found no changes (4) (Figure 1).

## 4. Discussion

According to multinational guidelines, behavioral treatment should represent frontline therapy in the case of OAB. Magnetic stimulation offers some theoretical advantages over all other behavioral treatment options, including PFMT, functional electrical stimulation, and biofeedback. Compared to electrical stimulation and biofeedback, MS is a type of passive rehabilitation that is performed passively and with no need for vaginal probes, which may cause discomfort among patients due to potentially compromised vaginal habitability. Moreover, there is no need for the patient to get undressed during treatment [65]. In fact, patients sit in an ergonomic chair, as it is equipped with a height-adjustable backrest, so it is possible to enjoy complete comfort and relaxation during every session. Other negative aspects related to electrical stimulation and biofeedback involve low adherence to therapy and the documented affection caused by tissue impedance to electrical current, which instead does not impact the conduction of electromagnetic energy. Low compliance with first-line treatments concerns even PFMT, as many patients may not be able to perform correct and consistent contractions and training of pelvic floor muscles, leading to poor results in terms of symptom improvement [66].

On the contrary, MS is a first-line non-invasive, standardizable, and with no adverse effects treatment for OAB [67]. Specifically, FMS is the newest innovation in MS technology. This novel device works by inducing an electric current and provoking passive, vigorous contractions of pelvic floor muscle (PFM), targeting neuromuscular tissue. Magnetic stimulation-related electric currents cause neuron depolarization, inducing concentric contractions and lifting all PFMs, which result in being deeply stimulated as well as neuromuscular control being regenerated [68,69]. Moreover, the entire process leads to a modification of the muscular structure as fibers tend to become hypertrophic and hyperplasic. Leone et al. have previously documented this modification on abdominal muscles in 15 patients who underwent FMS, reporting an augmented muscular thickness 1 month after treatment in the four areas that had been targeted by therapy: upper, lower, lateral abdomen, and rectus abdominis diastasis [70]. Moreover, as a consequence of FMS, Frigerio et al. have reported substantial rhabdosphincter muscle hypertrophy, consisting of a 15.4% augmentation in muscular total volume [56]. This is particularly relevant, considering that strengthening the bladder outlet muscles is supposed to be effective in suppressing urgency. This makes FMS a promising device to treat urge urinary incontinence that can eventually be used as exclusive therapy or together with other pharmacological or physical procedures.

Our experience aimed to prospectively compare the short-term outcomes of FMS in patients with urge urinary incontinence and no other ongoing treatments, evaluating urinary symptoms at baseline and at the end of treatment. According to PGI-I scores, 42 (73.7%) women referred to some kind of improvement in their OAB symptoms after 8 sessions of FMS. Considering QoL outcomes, after the MS sessions, we reported a statistically significant decrease in the IIQ7 scores (a troubling amount of leakages) compared to baseline. In the same way, a statistically significant decrease in the ICIQ-UI SF scores was observed. Patients’ sexual function was assessed by the FSFI-19 questionnaire, and this study results demonstrate an improvement related to the impact of urge incontinence on patients’ sexual lives.

The effectiveness of FMS for mixed/stress and urge incontinence was previously highlighted by other studies; Lopopolo et al. investigated the impact of FMS on mixed urinary incontinence in 50 patients. Women underwent 6 sessions of treatment with the Dr. ARNOLD system (DEKA M.E.L.A. Calenzano, Italy), divided into two of them per week for 3 weeks, for approximately 30 min. Multiple questionnaires were used for the assessment of the urinary symptoms before, within, and 3 months after the treatment period: ICIQ-UI-SF, Incontinence Questionnaire Overactive Bladder Module (ICIQ-OAB), and IIQ-7. Data suggested that FMS technology could reduce mixed urinary incontinence symptoms for all the patients in this study, reflecting an improvement in their QoL [71]. Biondo et al. analyzed 46 female patients reporting urge urinary incontinence who underwent a total of 8 treatment sessions performed twice a week for 4 consecutive weeks for 28 min. Immediately before each treatment and up to 3 months of follow-up, two questionnaires, IIQ-7 and ICIQ-OAB, were used. They concluded that the protocol used led to a decrease in urge incontinence complaints, achieving good results and improving patients’ QoL without risk [72]. In a prospective study, Doğanay et al. documented the long-term effects induced by extracorporeal magnetic stimulation on 69 patients with urge incontinence who performed 16 sessions of treatment. At 6-month follow-up, patients in both groups had a significant improvement in QoL, related to a significant decrease in leakage episodes and daily pad use [73].

Strengths of our study involve the prospective design in which a clinical urogynecological evaluation was offered to each patient, the exhaustive assessment of benefits—in particular, subjective and objective curative rate and several validated QoL questionnaires—and the low percentage of lost patients at follow-up (1.7%, 1 out of 58). Limitations are represented by the lack of a control group and a small sample size.

## 5. Conclusions

Our experience demonstrated that FMS constitutes a safe and effective option for treating naive and refractory urge urinary incontinence in terms of objective and subjective cure rate, with no adverse effects described as well as a high level of acceptance for the patients.

## Figures and Tables

**Table 1:** Baseline population characteristics. ICIQ-UI SF: International Consultation on Incontinence Questionnaire–Urinary Incontinence Short Form; FSFI-19: Female Sexual Function Index; IIQ-7: Incontinence Impact Questionnaire. Continuous data as mean ± standard deviation. Non-continuous data as absolute (relative) frequency.

Age (years)	64.6 ± 11.5
Parity (*n*)	1.6 ± 1.0
Previous second-line treatments (*n*)	0.7 ± 1.0
Previous third-line treatments (*n*)	0.1 ± 0.3
Total previous second- and third-line treatments (*n*)	0.8 ± 1.0
ICIQ-UI SF score (T0)	11.7 ± 4.9
IIQ-7 score (T0)	41.7 ± 23.0
FSFI-19 score (T0)	9.2 ± 10.4

**Table 2:** Baseline (T0) versus end-of-treatment (T1) comparison. Data are reported as medians and interquartile ranges. ICIQ-UI SF: International Consultation on Incontinence Questionnaire–Urinary Incontinence Short Form; FSFI-19: Female Sexual Function Index; IIQ-7: Incontinence Impact Questionnaire; PGI-I: Patient Global Impression of Improvement. n/A: not applicable.

Questionnaire	Baseline (T0)	End of Treatment (T1)	*p*-Value
IIQ-7	33 [27.5–55.0]	27.5 [11.0–44.0]	<0.001
ICIQ-UI SF	13 [8–16]	8 [6–13]	<0.001
FSFI-19	1.2 [1.2–18.5]	2.7 [1.2–21.4]	<0.001
PGI-I	n/A	3 [2–4]	n/A

## Data Availability

The data described in the current study are accessible on demand from the corresponding author.

## References

[1] Good M.M., Solomon E.R. (2019). Pelvic Floor Disorders. Obstet. Gynecol. Clin. N. Am..

[2] Wu J.M., Vaughan C.P., Goode P.S., Redden D.T., Burgio K.L., Richter H.E., Markland A.D. (2014). Prevalence and trends of symptomatic pelvic floor disorders in U.S. women. Obstet. Gynecol..

[3] Hallock J.L., Handa V.L. (2016). The Epidemiology of Pelvic Floor Disorders and Childbirth: An Update. Obstet. Gynecol. Clin. N. Am..

[4] Handa V.L., Blomquist J.L., McDermott K.C., Friedman S., Muñoz A. (2012). Pelvic floor disorders after vaginal birth: Effect of episiotomy, perineal laceration, and operative birth. Obstet. Gynecol..

[5] Blomquist J.L., Muñoz A., Carroll M., Handa V.L. (2018). Association of Delivery Mode with Pelvic Floor Disorders after Childbirth. JAMA.

[6] Tsakiridis I., Mamopoulos A., Athanasiadis A., Dagklis T. (2018). Obstetric Anal Sphincter Injuries at Vaginal Delivery: A Review of Recently Published National Guidelines. Obstet. Gynecol. Surv..

[7] Pergialiotis V., Vlachos D., Protopapas A., Pappa K., Vlachos G. (2014). Risk factors for severe perineal lacerations during childbirth. Int. J. Gynaecol. Obstet..

[8] Barba M., Bernasconi D.P., Manodoro S., Frigerio M. (2022). Risk factors for obstetric anal sphincter injury recurrence: A systematic review and meta-analysis. Int. J. Gynaecol. Obstet..

[9] Doumouchtsis S.K., de Tayrac R., Lee J., Daly O., Melendez-Munoz J., Lindo F.M., Cross A., White A., Cichowski S., Falconi G. (2023). An International Continence Society (ICS)/International Urogynecological Association (IUGA) joint report on the terminology for the assessment and management of obstetric pelvic floor disorders. Int. Urogynecol. J..

[10] Manodoro S., Spelzini F., Cesana M.C., Frigerio M., Maggioni D., Ceresa C., Penati C., Sicuri M., Fruscio R., Nicolini G. (2017). Histologic and metabolic assessment in a cohort of patients with genital prolapse: Preoperative stage and recurrence investigations. Minerva Ginecol..

[11] Kerkhof M.H., Ruiz-Zapata A.M., Bril H., Bleeker M.C., Belien J.A., Stoop R., Helder M.N. (2014). Changes in tissue composition of the vaginal wall of premenopausal women with prolapse. Am. J. Obstet. Gynecol..

[12] Johnston S.L. (2019). Pelvic Floor Dysfunction in Midlife Women. Climacteric.

[13] Mannella P., Palla G., Bellini M., Simoncini T. (2013). The female pelvic floor through midlife and aging. Maturitas.

[14] DeLancey J.O., Trowbridge E.R., Miller J.M., Morgan D.M., Guire K., Fenner D.E., Weadock W.J., Ashton-Miller J.A. (2008). Stress urinary incontinence: Relative importance of urethral support and urethral closure pressure. J. Urol..

[15] Milani R., Frigerio M., Vellucci F.L., Palmieri S., Spelzini F., Manodoro S. (2018). Transvaginal native-tissue repair of vaginal vault prolapse. Minerva Ginecol..

[16] Milani R., Frigerio M., Spelzini F., Manodoro S. (2017). Transvaginal uterosacral ligament suspension for posthysterectomy vaginal vault prolapse repair. Int. Urogynecol. J..

[17] Amundsen C.L., Flynn B.J., Webster G.D. (2003). Anatomical Correction of Vaginal Vault Prolapse by Uterosacral Ligament Fixation in Women Who Also Require a Pubovaginal Sling. J. Urol..

[18] Frigerio M., Manodoro S., Cola A., Palmieri S., Spelzini F., Milani R. (2019). Risk factors for persistent, de novo and overall overactive bladder syndrome after surgical prolapse repair. Eur. J. Obstet. Gynecol. Reprod. Biol..

[19] Diez-Itza I., Aizpitarte I., Becerro A., Sarasqueta C. (2009). Incidence of overactive bladder after vaginal hysterectomy and associated repairs for pelvic organ prolapse. Gynecol. Obstet. Investig..

[20] Abrams P., Cardozo L., Fall M., Griffiths D., Rosier P., Ulmsten U., Van Kerrebroeck P., Victor A., Wein A. (2002). The standardization of terminology of lower urinary tract function: Report from the standardization sub-committee of the International Continence Society. Neurourol. Urodyn..

[21] Stewart W., Van Rooyen J., Cundiff G., Abrams P., Herzog A., Corey R., Hunt T., Wein A. (2003). Prevalence and burden of overactive bladder in the United States. World J. Urol..

[22] Frigerio M., Barba M., Cola A., Braga A., Celardo A., Munno G.M., Schettino M.T., Vagnetti P., De Simone F., Di Lucia A. (2022). Quality of life, psychological wellbeing, and sexuality in women with urinary incontinence—Where are we now: A narrative review. Medicina.

[23] Verbeek M., Hayward L. (2019). Pelvic Floor Dysfunction and Its Effect on Quality of Sexual Life. Sex. Med. Rev..

[24] Yamaguchi O., Honda K., Nomiya M., Shishido K., Kakizaki H., Tanaka H., Yamanishi T., Homma Y., Takeda M., Araki I. (2007). Defining overactive bladder as hypersensitivity. Neurourol. Urodyn..

[25] Manodoro S., Barba M., Locatelli L., Palmieri S., Marino G., Frigerio M. (2020). Urodynamic predictors of deo overactive bladder after single-incision sling. Int. J. Gynaecol. Obstet..

[26] Hashim H., Abrams P. (2006). Is the bladder a reliable witness for predicting detrusor overactivity?. J. Urol..

[27] Digesu G.A., Khullar V., Cardozo L., Salvatore S. (2003). Overactive bladder symptoms: Do we need urodynamics?. Neurourol. Urodyn..

[28] Rosier P.F., Giarenis I., Valentini F.A., Wein A., Cardozo L. (2014). Do patients with symptoms and signs of lower urinary tract dysfunction need a urodynamic diagnosis? ICI-RS 2013. Neurourol. Urodyn..

[29] Frigerio M., Barba M., Marino G., Volontè S., Melocchi T., De Vicari D., Torella M., Salvatore S., Braga A., Serati M. (2022). Coexistent detrusor overactivity-underactivity in patients with pelvic floor disorders. Healthcare.

[30] D’Alessandro G., Palmieri S., Cola A., Barba M., Manodoro S., Frigerio M. (2022). Detrusor underactivity prevalence and risk factors according to different definitions in women attending urogynecology clinic. Int. Urogynecol. J..

[31] Frigerio M., Barba M., Cola A., Volontè S., Marino G., Regusci L., Sorice P., Ruggeri G., Castronovo F., Serati M. (2022). The learning curve of urodynamics for the evaluation of lower urinary tract symptoms. Medicina.

[32] Frigerio M., Manodoro S., Cola A., Palmieri S., Spelzini F., Milani R. (2018). Detrusor underactivity in pelvic organ prolapse. Int. Urogynecol. J..

[33] Frigerio M., Barba M., Cola A., Spelzini F., Milani R., Manodoro S. (2023). Coexisting overactive-underactive bladder and detrusor overactivity-underactivity in pelvic organ prolapse. Int. J. Gynaecol. Obstet..

[34] Roosen A., Chapple C.R., Dmochowski R.R., Fowler C.J., Gratzke C., Roehrborn C.G., Stief C.G., Andersson K.-E. (2009). A refocus on the bladder as the originator of storage lower urinary tract symptoms: A systematic review of the latest literature. Eur. Urol..

[35] Apostolidis A., Averbeck M.A., Sahai A., Rahnama’I M.S., Anding R., Robinson D., Gravas S., Dmochowski R. (2017). Can we create a valid treatment algorithm for patients with drug-resistant overactive bladder (OAB) syndrome or detrusor overactivity (DO)? Results from a think tank (ICI-RS 2015). Neurourol. Urodyn..

[36] Hubeaux K., Deffieux X., Ismael S.S., Raibaut P., Amarenco G. (2007). Autonomic nervous system activity during bladder filling assessed by heart rate variability analysis in women with idiopathic overactive bladder syndrome or stress urinary incontinence. J. Urol..

[37] Hubeaux K., Deffieux X., Raibaut P., Le Breton F., Jousse M., Amarenco G. (2011). Evidence for autonomic nervous system dysfunction in females with idiopathic overactive bladder syndrome. Neurourol. Urodyn..

[38] Bunn F., Kirby M., Pinkney E., Cardozo L., Chapple C., Chester K., Cruz F., Haab F., Kelleher C., Milsom I. (2015). Is there a link between overactive bladder and the metabolic syndrome in women? A systematic review of observational studies. Int. J. Clin. Pract..

[39] Hsu L.N., Hu J.C., Chen P.Y., Lee W.C., Chuang Y.C. (2022). Metabolic syndrome and overactive bladder syndrome may share common pathophysiologies. Biomedicines.

[40] Saratlija Novakovic Z., Tesija R.A., Puljak L. (2017). Association between metabolic syndrome and overactive bladder: A case-control study. Scand. J. Urol..

[41] Hanna-Mitchell A.T., Robinson D., Cardozo L., Everaert K., Petkov G.V. (2016). Do we need to know more about the effects of hormones on lower urinary tract dysfunction? ICI-RS 2014. Neurourol. Urodyn..

[42] Klausner A.P., Steers W.D. (2004). Corticotropin-releasing factor: A mediator of emotional influences on bladder function. J. Urol..

[43] Malykhina A.P., Wyndaele J.J., Andersson K.E., De Wachter S., Dmochowski R.R. (2012). Do the urinary bladder and large bowel interact, in sickness or in health? ICI-RS 2011. Neurourol. Urodyn..

[44] Panicker J.N., Marcelissen T., von Gontard A., Vrijens D., Abrams P., Wyndaele M. (2019). Bladder-bowel interactions: Do we understand pelvic organ cross-sensitization? International Consultation on Incontinence Research Society (ICI-RS) 2018. Neurourol. Urodyn..

[45] Aragón I.M., Herrera-Imbroda B., Queipo-Ortuño M.I., Castillo E., Del Moral J.S.-G., Gómez-Millán J., Yucel G., Lara M.F. (2018). The urinary tract microbiome in health and disease. Eur. Urol. Focus.

[46] Karstens L., Asquith M., Davin S., Stauffer P., Fair D., Gregory W.T., Rosenbaum J.T., McWeeney S.K., Nardos R. (2016). Does the Urinary Microbiome Play a Role in Urgency Urinary Incontinence and Its Severity?. Front. Cell. Infect. Microbiol..

[47] Peyronnet B., Mironska E., Chapple C., Cardozo L., Oelke M., Dmochowski R., Amarenco G., Gamé X., Kirby R., Van Der Aa F. (2019). A Comprehensive Review of Overactive Bladder Pathophysiology: On the Way to Tailored Treatment. Eur. Urol..

[48] Jayarajan J., Radomski S.B. (2013). Pharmacotherapy of overactive bladder in adults: A review of efficacy, tolerability, and quality of life. Res. Rep. Urol..

[49] Moyson J., Legrand F., Vanden Bossche M., Quackels T., Roumeguère T. (2017). Efficacy and safety of available therapies in the management of idiopathic overactive bladder: A systematic review of the literature. Prog. Urol..

[50] Lightner D.J., Gomelsky A., Souter L., Vasavada S.P. (2019). Diagnosis and Treatment of Overactive Bladder (Non-Neurogenic) in Adults: AUA/SUFU Guideline Amendment 2019. J. Urol..

[51] Imran M., Najmi A.K., Tabrez S. (2013). Mirabegron for overactive bladder: A novel, first-in-class β3-agonist therapy. Urol. J..

[52] AChen H.L., Chen T.C., Chang H.M., Juan Y.S., Huang W.H., Pan H.F., Chang Y.C., Wu C.M., Wang Y.L., Lee H.Y. (2018). Mirabegron is an alternative to antimuscarinic agents for overactive bladder without higher risk in hypertension: A systematic review and meta-analysis. World J. Urol..

[53] Lin C.T., Chiang B.J., Liao C.H. (2020). Perspectives of medical treatment for overactive bladder. Urol. Sci..

[54] Lo C.W., Wu M.Y., Yang S.S., Jaw F.S., Chang S.J. (2020). Comparing the Efficacy of OnabotulinumtoxinA, Sacral Neuromodulation, and Peripheral Tibial Nerve Stimulation as Third-Line Treatment for the Management of Overactive Bladder Symptoms in Adults: Systematic Review and Network Meta-Analysis. Toxins.

[55] Niu H.L., Ma Y.H., Zhang C.J. (2018). Comparison of OnabotulinumtoxinA versus sacral neuromodulation for refractory urinary urge incontinence: A systematic review and meta-analysis of randomized controlled trials. Int. J. Surg..

[56] Frigerio M., Barba M., Cola A., Marino G., Volontè S., Melocchi T., De Vicari D., Maruccia S. (2023). Flat Magnetic Stimulation for Stress Urinary Incontinence: A Prospective Comparison Study. Bioengineering.

[57] Galloway N.T.M., El-Galley R.E., Sand P.K., Appell R.A., Russell H.W., Carlan S.J. (1999). Extracorporeal magnetic innervation therapy for stress urinary incontinence. Urology.

[58] Braga A., Castronovo F., Caccia G., Papadia A., Regusci L., Torella M., Salvatore S., Scancarello C., Ghezzi F., Serati M. (2022). Efficacy of 3 Tesla Functional Magnetic Stimulation for the Treatment of Female Urinary Incontinence. J. Clin. Med..

[59] Lukanović D., Kunič T., Batkoska M., Matjašič M., Barbič M. (2021). Effectiveness of Magnetic Stimulation in the Treatment of Urinary Incontinence: A Systematic Review and Results of Our Study. J. Clin. Med..

[60] Barba M., Cola A., Rezzan G., Costa C., Melocchi T., De Vicari D., Terzoni S., Frigerio M., Maruccia S. (2023). Flat Magnetic Stimulation for Stress Urinary Incontinence: A 3-Month Follow-Up Study. Healthcare.

[61] Tubaro A., Zattoni F., Prezioso D., Scarpa R.M., Pesce F., Rizzi C.A., Santini A.M., Simoni L., Artibani W., The Flow Study Group (2006). Italian validation of the international consultation on incontinence questionnaires. BJU Int..

[62] Filocamo M.T., Serati M., Lizi V., Costantini E., Milanesi M., Pietropaolo A., Polledro P., Gentile B., Maruccia S., Fornia S. (2014). The Female Sexual Function Index (FSFI): Linguistic validation of the Italian version. J. Sex. Med..

[63] Monticone M., Frigau L., Mola F., Rocca B., Giordano A., Foti C., Franchignoni F. (2021). Italian versions of the Urogenital Distress Inventory-6 and Incontinence Impact Questionnaire-7: Translation and validation in women with urinary incontinence. Disabil. Rehabil..

[64] Srikrishna S., Robinson D., Cardozo L. (2010). Validation of the patient global impression of improvement (PGI-I) for urogenital prolapse. Int. Urogynecol. J..

[65] Takahashi S., Kitamura T. (2003). Overactive bladder: Magnetic versus electrical stimulation. Curr. Opin. Obstet. Gynecol..

[66] Greer J.A., Arya L.A., Smith A.L. (2013). Urinary incontinence: Diagnosis and treatment in the elderly. Curr. Transl. Geriatr. Exp. Gerontol. Rep..

[67] Pavčnik M., Antić A., Lukanović A., Krpan Ž., Lukanović D. (2023). Evaluation of Possible Side Effects in the Treatment of Urinary Incontinence with Magnetic Stimulation. Medicina.

[68] Sun K., Zhang D., Wu G., Wang T., Wu J., Ren H., Cui Y. (2021). Efficacy of magnetic stimulation for female stress urinary incontinence: A meta-analysis. Ther. Adv. Urol..

[69] APeng L., Zeng X., Shen H., Luo D.Y. (2019). Magnetic stimulation for female patients with stress urinary incontinence, a meta-analysis of studies with short-term follow-up. Medicine.

[70] Leone A., Piccolo D., Conforti C., Pieri L., Fusco I. (2021). Evaluation of safety and efficacy of a new device for muscle toning and body shaping. J. Cosmet. Dermatol..

[71] Lopopolo G., Salsi B., Banfi A., Isaza P.G., Fusco I. (2022). Is It Possible to Improve Urinary Incontinence and Quality of Life in Female Patients? A Clinical Evaluation of the Efficacy of Top Flat Magnetic Stimulation Technology. Bioengineering.

[72] Biondo A., Gonzalez Isaza P., Fusco I. (2022). Efficacy of top flat magnetic stimulation technology for female stress and urge urinary incontinence: A clinical evaluation. World J. Nephrol. Urol..

[73] Doğanay M., Kılıç S., Yılmaz N. (2010). Long-term effects of extracorporeal magnetic innervations in the treatment of women with urinary incontinence: Results of 3-year follow-up. Arch. Gynecol. Obstet..

